# Perspiration

**Published:** 1871-02

**Authors:** 


PERSPIRATION.—The amount of liquid matter which passes through the microscopical tubes of the skin in twenty-four hours, in an adult person of sound health, is about sixteen fluid ounces, or one pint. One ounce of the sixteen is solid matter, made up of organic and inorganic substances, which, if allowed to remain in the system for a brief space of time, would cause death. The rest is water. Beside the water and solid matter, a large amount of carbonic acid, a gaseous body, passes through the tubes; so we cannot fail to understand that they are active workers, and also we cannot fail to see the importance of keeping them in perfect working order, removing obstructions by frequent application of water, or by some other means. Suppose we obstruct the functions of the skin perfectly, by varnishing a person completely with a compound impervious to moisture. How long will he live? Not over six hours. The experiment was once tried on a child at Florence. Pope Leo, the Tenth, on the occasion of his accession to the papal chair, wished to have a living figure to represent the Golden Age, and so he gilded a poor child all over with varnish and gold leaf. The child died in a few hours. If the fur of a rabbit or the skin of a pig be covered with a solution of india-rubber in naphtha, the animal ceases to breathe in a couple of hours.—*Journal of Chemistry.*

